# Supplementary figures and images for: BC-store: A program for MGISEQ barcode sets analysis
Source: PLoS One. 2021 Mar 1;16(3):e0247532. doi: 10.1371/journal.pone.0247532 (PMC7920359; doi:10.1371/journal.pone.0247532)

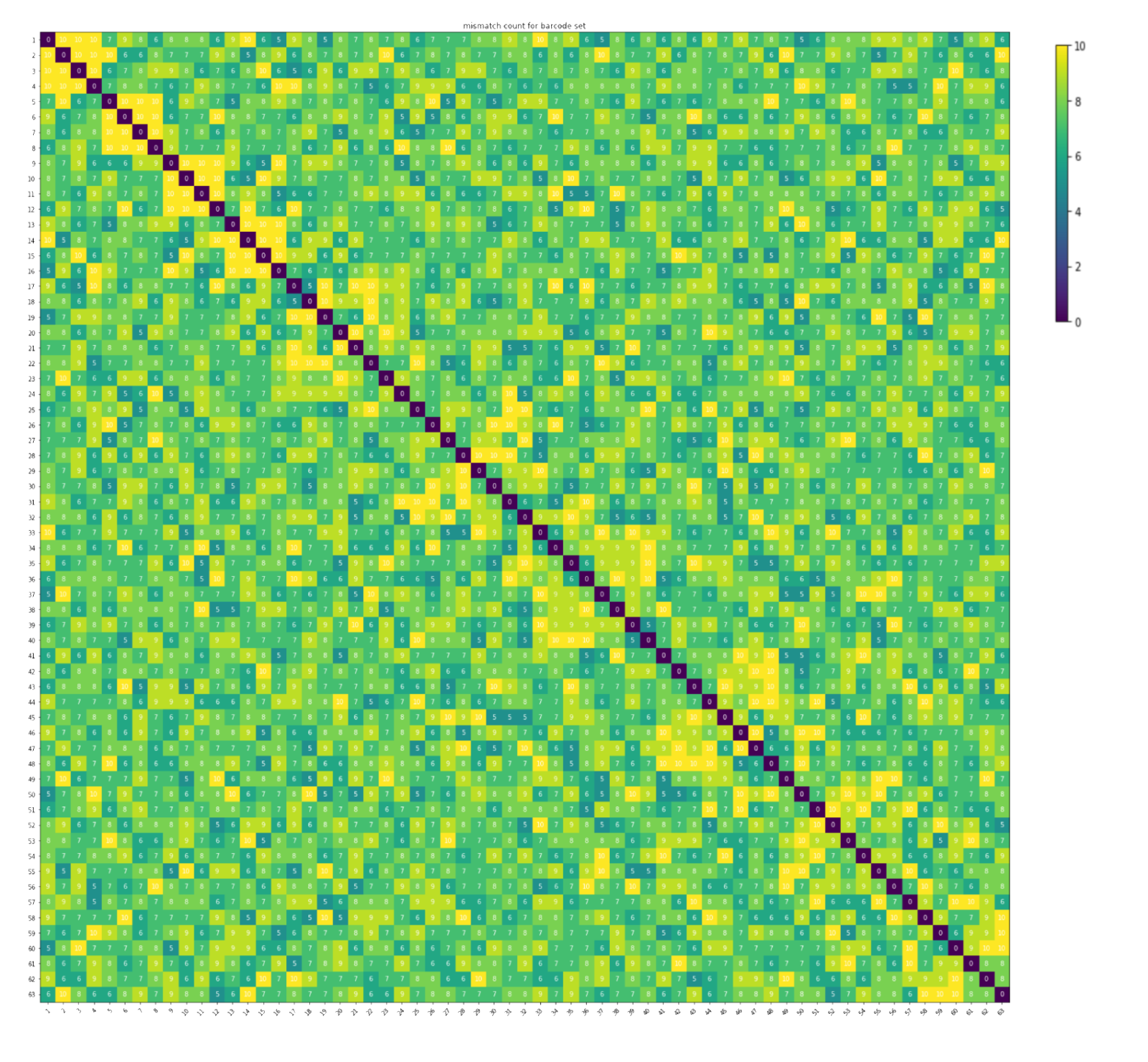

Supplement: S1 Fig — The number and color indicate the number of different nucleotides in the barcodes. The greater the difference, the more likely it is that samples with these barcodes will not merge during sequencing. (TIF) [file pone.0247532.s001.tif]

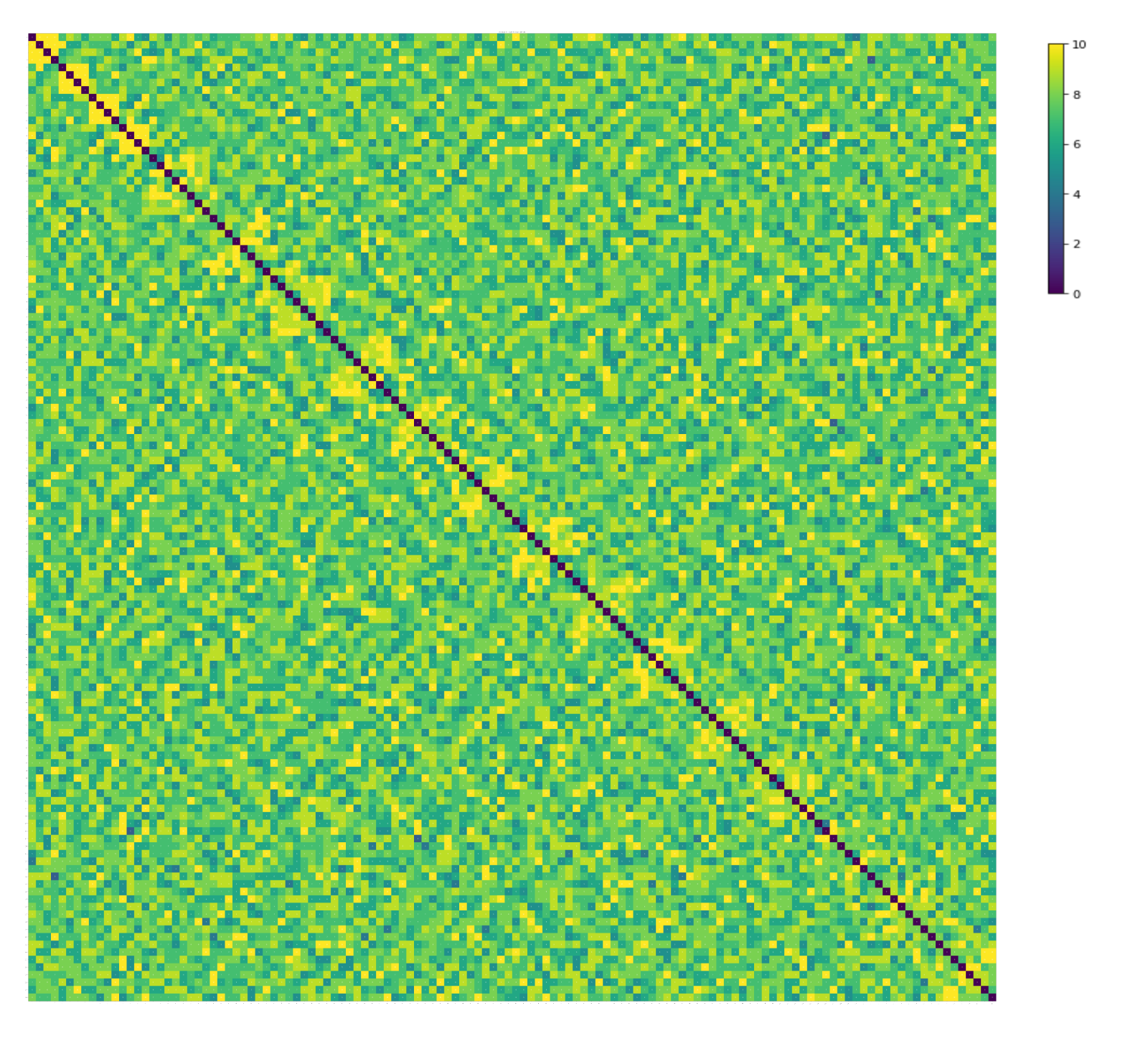

Supplement: S2 Fig — The number and color indicate the number of different nucleotides in the barcodes. (TIF) [file pone.0247532.s002.tif]
