# Supplementary material for: BC-store: A program for MGISEQ barcode sets analysis
Source: PLoS One. 2021 Mar 1;16(3):e0247532. doi: 10.1371/journal.pone.0247532 (PMC7920359; doi:10.1371/journal.pone.0247532)
Supplement: S1 Text — Instruction for user. (DOCX) [file pone.0247532.s003.docx]

Architecture

BC-store is available in two versions: desktop for Windows and command line for operating systems with pre-installed Python3, available for download at <https://github.com/genomecenter/BC-store>.

How it works from the command line

The program is launched by running it from the command line:

python3 [path to bc-store_script.py] [command] [options by whitespace]

There are 4 main commands:

check_set – checks the current set based on the proportions according to the selected criterion

add_to_set – selects the desired number of barcodes and adds to the existing set, taking into account the proportions according to the selected criterion from the selected set of barcodes

help – help with the examples of commands and explanation of options and format for entering parameters

MGI_sets – displays successful sets of barcodes according to the MGI manual

After running the check_set commands and if the set selection is successful, the user will be provided with a graph by the add_to_set command. To run a new command, user needs to close the drawing and save it if necessary before closing. To use the script, some standard packages specified at the beginning of the python3 file are required. Rate can be a fractional number and is written in the command through a dot like “2.4”.

Examples of how BC-store works from the command line for “check_set” in the case of equal and unequal proportions of barcodes mixing in a set are shown in Fig 1.

The first one is activated by the command:

python3 bc-store_script.py check_set set=13,14,15,16 rate=1,1,1,1 criteria=strong

or

python3 bc-store_script.py check_set set=13,14,15,16 rate=equal criteria=strong

The second one is activated by the command:

python3 bc-store_script.py check_set set=13,14,15,16 rate=1,4,1,4 criteria=strong

Figure 1. Example of BC-store operation from the command line in case of equal (A) and unequal (B) proportions of barcodes mixed in a set.

For “add_to_set” command example:

python3 bc-store_script.py add_to_set current_set=1,2,3,4 how_many_add=2 rate_with_add=1,2,1,2,3,4 criteria=lite from_barcodes=13,14,15,16

For “help” and “MGI_sets” commands example respectively:

python3 bc-store_script.py help

python3 bc-store_script.py MGI_sets

How it works on the desktop version

The desktop version with the numbering of input fields and buttons is illustrated in Fig 2. When user starts the desktop version, an additional window opens where errors are displayed so that user does not need to close it for the program to work correctly. The main areas of work are set analysis (sections on Fig 2 : 1, 2, 3), set selection (sections on Fig 2: 1, 5, 6, 7, 9, 10) and output of basic sets (section on Fig 2: 8). After clicking the "Click to analyse set" button and, if there are additional set barcodes, when clicking the "Click to analyze adding" button, the user will be provided with a graph. User should not press buttons 3 and 10 until user has filled in all the required fields. The program presents the output information on user-defined barcodes, mixing proportions, values of nucleotide representation, and the main information on whether this set meets the strong and lite criteria (Fig 3).


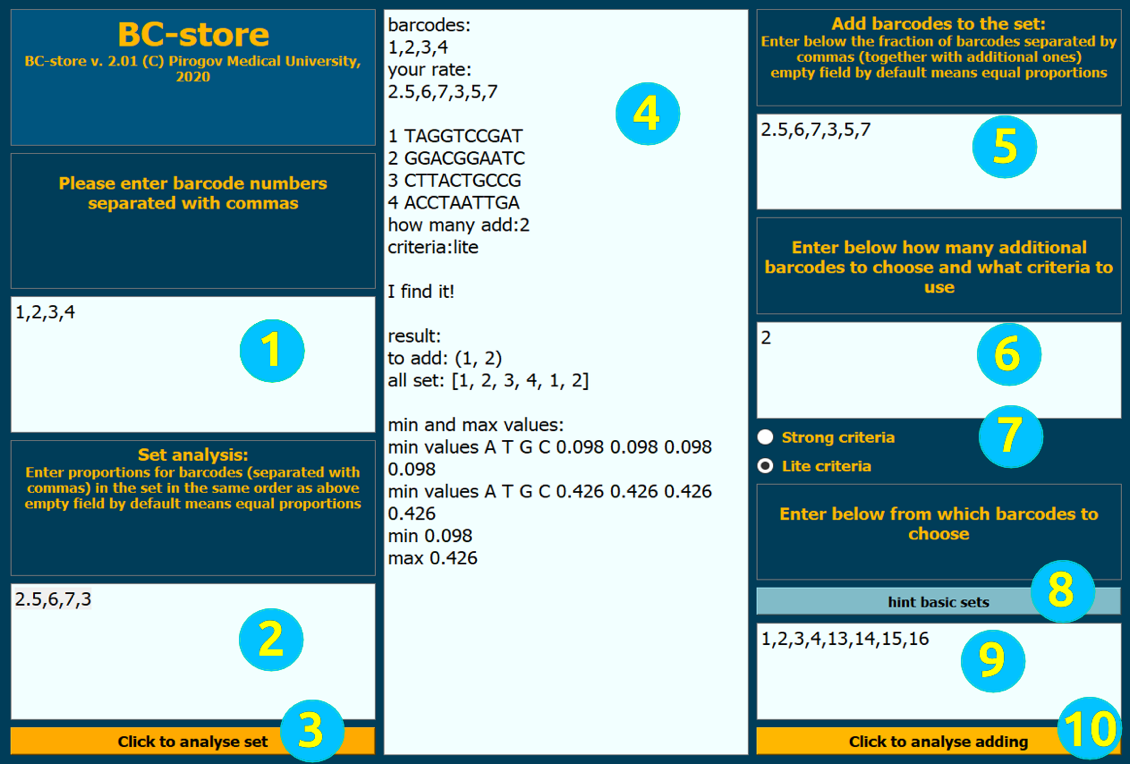


Figure 2. Layout of the desktop version with numbering of input fields and buttons. 1 – enter the current set, 2 – enter set proportions, 3 – set analysis button, 4 – output field, 5 – input proportions of the current set with the new barcodes, 6 – enter the number of new barcodes, 7 – select criteria, 8 – tip for base sets, 9 – input the barcode to choose from, 10 – start analysis of set selection.

Figure 3. A – an example of how BC-store works in case of equal proportions of barcodes in a set. In the case of equal proportions, the field for entering shares should be left free. B – an example of how BC-store works if the other barcodes are added to the set.
